# Reliability and Validity of the Swedish Version of the Parents' Postoperative Pain Measure (PPPM‐S): A Cross‐Sectional Psychometric Study

**DOI:** 10.1111/scs.70236

**Published:** 2026-03-27

**Authors:** C. Hermansson, I. Thylén, B. Grossmann

**Affiliations:** ^1^ Department of Anesthesia and Intensive Care Region Östergötland Linköping Sweden; ^2^ Department of Health, Medicine, and Caring Sciences Linköping University Linköping Sweden; ^3^ Clinical Department of Cardiology Region Östergötland Linköping Sweden; ^4^ Department of Health Blekinge Institute of Technology Karlskrona Sweden

**Keywords:** paediatric, pain assessment, parents, postoperative pain, PPPM, psychometric

## Abstract

**Background:**

As paediatric day surgery becomes increasingly common, postoperative care is transferred to the home setting where parents play a central role in assessing and managing their child's postoperative pain. No validated Swedish instrument currently exists to support parents in evaluating their child's pain at home.

**Aim:**

To assess the reliability and validity of the Swedish translation of the PPPM‐S in children aged 2–12 years during the first two postoperative days.

**Method:**

The instrument was earlier translated from English into Swedish in accordance with the WHO Guidelines for translation and adaptation of an instrument. A backward‐forward translation was done with a bilingual expert panel, and cognitive interviews were done in the target population. This study was conducted at three Swedish hospitals between 2022 and 2025 involving 80 parents of children aged 2–12 years who underwent day surgery. Parents completed the PPPM‐S on postoperative days 1 and 2. To evaluate the accuracy of the instrument, the results were compared with scores from an established pain rating scale, the Coloured Analogue Scale (CAS).

**Findings:**

PPPM‐S demonstrated good psychometric properties: good internal consistency (Cronbach's alpha 0.842–0.851) and satisfactory criterion validity demonstrated by strong correlations with CAS (Spearman's rho = 0.683–0.630, *p* < 0.01). ROC analysis identified 5/15 as an optimal cut‐off, with acceptable sensitivity and specificity. Parents reported high levels of satisfaction and found it easy to use at home.

**Conclusion:**

The PPPM‐S is a valid and practical tool for assessing children's postoperative pain at home. It can help parents better understand and evaluate their child's pain, potentially improving postoperative care in the home setting.

## 1 Introduction

Many surgeries today are performed as day surgeries, meaning patients do not need to stay overnight in the hospital. This shift is partly due to limited resources and reduced hospital bed capacity. Advances in surgical techniques and perioperative care have also contributed to enhanced postoperative recovery and fewer complications. This trend is seen not only in adults but also in children, where the number of day surgeries has increased in recent years in Sweden [1]. When a child is discharged on the same day as their surgery, the responsibility for postoperative care shifts to the parents or guardians. However, studies have shown that parents often find it challenging to assess their child's pain, and they tend to underestimate the pain level, resulting in inadequate pain management [2, 3]. Both MacLaren et al. and Makhlouf et al. [4, 5] have described how poorly managed or untreated pain in children can lead to various complications and trauma, including unnecessary fear and anxiety about future medical care. When the responsibility for assessing postoperative pain shifts from healthcare professionals to parents, it places a significant burden on them. Therefore, parents must be given appropriate tools and support to accurately evaluate their child's pain, ensuring effective pain management [2, 6].

Several instruments exist for measuring children's pain in Sweden, including the Coloured Analogue Scale (CAS), Face Pain Scale‐Revised (FPS‐R), Visual analogue scale (VAS), and the Face, Legs, Activity, Cry, Consolability (FLACC) scale [7, 8, 9]. However, these tools were developed for use in clinical settings by healthcare professionals and have not been validated for home use by parents [5]. The Parents' Postoperative Pain Measure (PPPM) was developed in Canada to help parents assess and manage their child's postoperative pain at home [10]. The PPPM focuses on observable changes in the child's behaviour after surgery to identify pain. It is currently the only validated instrument specifically designed for parents to assess their child's pain at home [5, 11]. The PPPM has been translated and validated in several languages, including Korean, Finnish, German, and Spanish [12, 13, 14, 15]. The aim of this study was to assess the reliability and validity of the Swedish translation of the PPPM‐S in children aged 2–12 years during the first two postoperative days.

## 2 Methods

### 2.1 Design

This study employed a cross‐sectional psychometric design to evaluate the reliability and validity of the Swedish version of the Parents' Postoperative Pain Measure (PPPM‐S). The design followed the STROBE statement for observational studies, with a focus on instrument validation in a clinical practice context [16].

### 2.2 Participants

The study was conducted in one university hospital, one regional hospital and one county hospital in Sweden between 1 March 2022, and 12 March 2025. Informed consent was obtained by staff at the operating wards of the three hospitals, who first provided the parents with both written and verbal information about the study. Only one parent per child was invited to participate. Parents who were unable to read or write in Swedish were excluded. The study included children aged 2 to 12 years who underwent day surgery, with the aim of assessing their postoperative pain at home on postoperative days 1 and 2. Of the 136 parents invited to participate, 84 (62%) returned completed forms. Four forms were excluded because they were not filled out correctly, resulting in a final sample of 80 participants.

### 2.3 Measurements

#### 2.3.1 Parents' Postoperative Pain Measure

The original PPPM consisted of 29 items derived from interviews with parents of children who had undergone surgery. During validation, Chambers et al. [10] identified 15 key items that formed the final PPPM scale, which was initially intended for children aged 7–12 years. The validity of the instrument was further supported by findings that PPPM scores decreased after analgesic administration [6]. Chambers et al. [17] later validated the PPPM for use in children aged 2–6 years. Parents are instructed to complete the measure between a specific time period (i.e., between breakfast and lunch, between lunch and supper, or supper and bedtime). Parents use the PPPM by observing their child during this period and answering ‘yes’ or ‘no’ to each item. The maximum score is 15, and a score of ≥ 6 indicates significant pain. This provides parents with a practical tool to identify their child's pain and administer the appropriate analgesics at the right time [3].

First, the Swedish version of the Parents' Postoperative Pain Measure (PPPM‐S) was translated and adapted to a Swedish context following the WHO's guidelines for the translation and adaptation of instruments [18, 19]. Two independent bilingual and bicultural translators, both native Swedish speakers, initially translated the instrument into Swedish. A backward‐forward translation was then performed by an expert group of paediatric healthcare professionals. Only minor adjustments were required and no major changes were made during the translation process. During the adaptation process, six interviews were conducted with parents of children aged 2–12 years who had undergone day surgery and would be at home during postoperative days 1 and 2. The interviews used the think‐aloud method to gain a deeper understanding of how respondents perceived the meaning of each item [20, 21]. These interviews confirmed that parents found all 15 items to be useful. One item, ‘Look more flushed than usual’, originally translated as ‘Ser mer rodnad ut än vanligt’ was modified to ‘Ser mer rodnad ut i ansiktet än vanligt’ to clarify the meaning. The final version of the PPPM‐S is presented in Table 1. A pilot study (*N* = 16) was conducted with a sample of the target population to test this version. The pilot study showed good validity, and that PPPM‐S has good psychometric properties.

**TABLE 1:** Original English version (PPPM) and Swedish translation (PPPM‐S).

English original version (PPPM)	Swedish translation (PPPM‐S)
1. Whine or complain more than usual?	1. Gnäller eller klagar mer än vanligt?
2. Cry more easily than usual?	2. Gråter lättare än vanligt?
3. Play less than usual?	3. Leker mindre än vanligt?
4. Not do the things s/he normally does?	4. Gör inte saker som han/hon brukar göra?
5. Act more worried than usual?	5. Verkar mer orolig än vanligt?
6. Act more quiet than usual?	6. Verkar mer stillsam än vanligt?
7. Have less energy than usual?	7. Orkar mindre än vanligt?
8. Refuse to eat?	8. Vägrar äta?
9. Eat less than usual?	9. Äter mindre än vanligt?
10. Hold the sore part of his/her body?	10. Håller om den ömmande delen av sin kropp?
11. Try not to bump the sore part?	11. Försöker att inte stöta emot eller använda den ömmande delen?
12. Groan or moan more than usual?	12. Jämrar eller stönar mer än vanligt?
13. Look more flushed than usual?	13. Ser mer rodnad ut i ansiktet än vanligt?
14. Want to be close to you more?	14. Vill vara nära dig mer än vanligt?
15. Take meds when normally refuse?	15. Tar medicin när han/hon normalt vägrar?

#### 2.3.2 Coloured Analogue Scale (CAS)

The CAS is an oblong plastic scale, measuring 18.5 cm in length and 4 cm in width. The front of the scale shows an increasing red intensity, visually representing the range from ‘no pain’ to ‘terrible pain’. The back of the scale is numbered from 0 to 10. A movable marker is used to estimate the pain level on the front of the scale, with the reading done by noting the corresponding value on the back. The CAS is validated for children aged 5 years and older [7]. The scale is considered user‐friendly, as it can be interpreted in three ways: by colour, length, and width [8]. This helps children to more easily understand how different positions on the scale represent varying levels of pain intensity [22]. Le May et al. [23] found that the CAS has high responsiveness and reliability, and it is recommended for assessing pain in children with acute pain.

#### 2.3.3 Satisfaction and Feasibility

After the parents assessed their child's pain on the second postoperative day, they answered four study‐specific questions about satisfaction and feasibility. They could answer ‘yes’ or ‘no’ to the following questions [1]. Is ‘yes’ and ‘no’ sufficient for answering? If not, what response option did you miss? [2] Did you have any particular situation that helped you assess your child's pain? If yes, what situation? [3] Was the tool easy to use for assessing your child's pain? [4] Do you feel that the form is practically feasible? In addition, the last two questions included space for additional comments.

### 2.4 Procedure

Parents were approached about the study after the surgery, once the child was settled and the parents were no longer solely focused on the immediate postoperative care. While the child was in the postoperative area, a nurse provided both written and verbal information about the study and invited parents to participate. Parents were instructed to adhere to the pain management guidelines provided by the surgeon. Parents who gave written consent received an envelope containing the PPPM‐S, the CAS, written information about the study, and a prepaid envelope for returning the completed materials. Each pre‐printed envelope and PPPM‐S questionnaire were assigned a number to serve as an anonymous code. Each department kept a list to document the respondent numbers and track which envelopes had been submitted. The questionnaire began with demographic questions about the child's age, gender, the type of surgery, and the parent's age, gender, and educational level. Parents assessed their child's pain once a day on postoperative days 1 and 2 at home, using the PPPM‐S to evaluate the child's behaviour. At the same time, a pain score was recorded using the CAS. Children aged 5 years and older completed the CAS themselves, while parents provided the ratings for younger children. This was found to be the easiest pain scale for parents to use at home for children unable to self‐report. After the second day's assessment, parents answered the four questions on satisfaction and feasibility. The completed forms were returned in the prepaid envelope. PPPM and CAS scores were calculated for each assessment and compiled with other raw data in an Excel file.

### 2.5 Statistical Analysis

Statistical analyses were performed using Jamovi [24]. Descriptive statistics (number and percentage) and measures of central tendency and dispersion (median and interquartile range, IQR) were calculated for background characteristics and postoperative pain levels. Cronbach's alpha was used to assess internal consistency across the 15 PPPM‐S items. Spearman's Rho examined criterion validity between PPPM‐S scores and CAS scores. A ROC analysis was performed to determine the optimal cut‐off score of the PPPM‐S. Differences in PPPM‐S scores and CAS between postoperative day 1 and day 2 were analysed using a paired *t*‐test or Wilcoxon signed‐rank test, depending on data distribution.

No a priori sample size calculation was conducted. However, the achieved sample size of 80 participants meets COSMIN's recommendations for instrument validation [25].

### 2.6 Ethics

The study was approved by the Swedish Ethical Review Authority (No. 2021–04862). In line with the Declaration of Helsinki, all participants received both verbal and written information about the study and were informed that they could withdraw at any time. Data were aggregated and presented at group level to ensure individual anonymity. Email contact was established with the developer of the PPPM to obtain permission and consent to validate the PPPM‐S.

## 3 Results

### 3.1 Demographic Data

Data were collected from 80 parents. Demographic information about the parents, children and type of surgery is shown in Table 2.

**TABLE 2:** Demographic characteristics.

Characteristics	*N*	%	Md	IQR
Parent's gender				
Female	64	80		
Male	16	20		
Parent's age			37	34–42
Parents education				
Elementary school	4	5		
High school	21	26		
University	55	69		
Child's gender				
Female	30	38		
Male	50	62		
Child's age			6.5	5–9.25
Surgery				
Orthopaedic	23	29		
Ear, Nose and Throat (ENT)	24	30		
Dental	3	4		
Oral and Maxillofacial	2	2		
Hand and Plastic	17	21		
General Surgery	11	14		

Abbreviations: IQ, interquartile range Q1‐Q3; Md, median.

### 3.2 Reliability

The internal consistency for the PPPM‐S on postoperative day 1 was α = 0.842, and on postoperative day 2 it was α = 0.851 (Table 3). Table 4 shows the Cronbach's alpha for each item if it were dropped, indicating strong overall internal consistency.

**TABLE 3:** Internal consistency and criterion validity.

	Cronbach's alpha (α)	Spearman's rho (r)	*p*
PPPM‐S Postoperative Day 1	0.851	0.683	< 0.001
PPPM‐S Postoperative Day 2	0.842	0.630	< 0.001

**TABLE 4:** Cronbach's alpha if item dropped.

PPPM‐S item	α if item dropped (Day 1)	α if item dropped (Day 2)
1. Whine or complain more than usual	0.816	0.829
2. Cry more easily than usual	0.832	0.845
3. Play less than usual	0.819	0.828
4. Not do the things s/he normally does	0.831	0.834
5. Act more worried than usual	0.834	0.852
6. Act more quiet than usual	0.816	0.830
7. Have less energy than usual	0.822	0.836
8. Refuse to eat	0.843	0.848
9. Eat less than usual	0.837	0.837
10. Hold the sore part of the body	0.835	0.850
11. Try not to bump the sore part	0.839	0.855
12. Groan or moan more than usual	0.824	0.839
13. Look more flushed in the face than usual	0.851	0.849
14. Want to be close to you more than usual	0.840	0.849
15. Take meds when normally refuse	0.841	0.847

### 3.3 Validity

The Spearman's rho correlation between the PPPM‐S and CAS was 0.683 (*p* < 0.001) on postoperative day 1 and 0.630 (*p* < 0.001) on postoperative day 2 (Table 3).

### 3.4 Psychometric Evaluation

The psychometric evaluation included an assessment of satisfaction and feasibility. Among respondents, 86.3% (*n* = 69) considered the binary response options (‘yes’ and ‘no’) sufficient for completing the PPPM‐S. Additionally, 93.8% (*n* = 75) reported that no relevant situations for assessing their child's pain were missing. Furthermore, 82.5% (*n* = 66) indicated that the PPPM‐S helped them assess their child's pain, and 98.8% (*n* = 79) found the instrument feasible for home use.

### 3.5 Postoperative Pain

Reported pain scores for the PPPM‐S and CAS are presented in Table 5.

**TABLE 5:** Postoperative pain scores.

	Postoperative Day 1 Md (IQR)	Postoperative Day 2 Md (IQR)	*p*
PPPM‐S	5 (1–8)	4 (2–5.25)	0.076
CAS	4 (1–7)	3 (1.5–5)	0.035

Abbreviations: IQ, interquartile range Q1‐Q3; Md, median.

ROC analysis supported a cut‐off score of 5/15 for identifying postoperative pain, with sensitivity of 75% and specificity of 78% on day 1, and sensitivity of 71% and specificity of 81% on day 2. The discriminatory ability was good, as reflected by AUC values of 0.777 (day 1) 0.816 and (day 2).

## 4 Discussion

The aim of this study was to validate the Swedish version of the PPPM, the PPPM‐S, for parents to use in assessing pain in children aged 2–12 years who had undergone day surgery. Cronbach's alpha ranged from 0.842 to 0.851, indicating high internal consistency and demonstrating good reliability of the PPPM‐S. These values are consistent with, or slightly higher than, those reported in previous validation studies of the instrument in other languages, where Cronbach's alpha typically ranged between 0.80 and 0.85 [12, 13, 14]. This supports the conclusion that the Swedish version performs comparably in terms of reliability.

In the original publications, the FPS was used as the gold standard when validating the PPPM [10, 17]. Other measurement instruments have also been used as gold standards in validating the PPPM in other languages. For example, the Finnish version used the VAS, while the German version used the FPS‐R and CHIPPS [12, 13]. In the Spanish validation, a 0–10 scale was used for parents rating children's pain up to 6 years old, with children aged 4–6 also rating themselves using the FPS, and children aged 6–12 using the 0–10 scale [14]. We chose the CAS because it is simple and easy to use, requiring no extensive review before use. It is validated for children aged 5 years and older, and in our study, those children rated their own pain. For younger children, there is no validated instrument for parents to use at home, and the CAS was considered the most straightforward and comprehensible alternative. In our study, the PPPM‐S demonstrated moderate to strong correlations with CAS, with Spearman's rho ranging from 0.630 to 0.683 across the two postoperative days. These findings are similar to those reported in earlier validation studies, where correlation coefficients between the PPPM and various established pain measures also fell within the moderate to strong range [12, 17]. This level of criterion validity supports the PPPM‐S as a credible measure of children's postoperative pain, particularly given that the CAS is a validated and well‐established tool within Swedish paediatric care and has shown responsiveness and reliability compared to other scales [23]. Our choice of CAS as a comparator also ensured broad applicability across age groups and yielded a complete dataset with no missing responses, thereby increasing the robustness of our findings.

An important contribution of this study is the evaluation of user satisfaction and feasibility, which is less commonly reported in similar validation research. A large majority of parents (98.8%) found the instrument feasible for home use, and over 80% stated that it helped them assess their child's pain. These findings underscore the clinical relevance of the PPPM‐S and suggest that it can serve not only as a valid measure but also as a supportive tool in the home setting, where parents often lack formal training in pain assessment. Notably, 86.3% of participants considered the binary response format sufficient, which aligns with the instrument's original design and intended ease of use [10].

Although a statistically significant reduction in pain was observed only in CAS scores from postoperative day 1 to day 2, the median PPPM‐S score also decreased from 5 to 4. This reflects expected clinical trajectories and may further support the sensitivity of the instrument to changes in pain over time. Similar patterns have been observed in previous studies, where pain typically diminishes within 48 h following minor surgical procedures [23]. This responsiveness is important for a parent‐administered tool intended to guide home‐based care decisions. Furthermore, a cut‐off score of ≥ 5 on the PPPM‐S emerged as a meaningful threshold at which parents are likely to need to administer analgesics to their child. Previous studies have suggested higher thresholds, typically ≥ 6, for identifying clinically relevant pain [10]. Our results therefore indicate that parental recognition of pain requiring intervention may occur at a slightly lower score, which could facilitate earlier and more consistent pain relief in the home setting. Early and consistent management of postoperative pain after surgery has been shown to be important in reducing the risk of persistent postsurgical pain [26].

### 4.1 Limitations

This study has some limitations [1]. Although the response rate (62%) was modest, it is relatively high for studies relying on postal questionnaires. Parents who did not respond may have differed in their experiences or perceptions of postoperative pain, which could affect the generalisability of the findings [2]. No a priori power analysis was conducted. However, the achieved sample size of 80 participants meets COSMIN's recommendations for instrument validation [25] and aligns with Bonett's guidance for estimating internal consistency in a 15‐item scale, assuming an expected Cronbach's alpha of 0.80 and a minimum acceptable threshold of 0.70 [3]. The use of three different hospitals in varied regions of Sweden enhances the ecological validity of the study. Nonetheless, certain limitations should be acknowledged. While our sample size meets COSMIN's recommendations for instrument validation [25], including a larger and more diverse population could have allowed for subgroup analyses, such as differences based on child age, type of procedure, or parental education level [4]. Construct validity was not assessed by comparing PPPM‐S scores with expected postoperative pain categories (mild, moderate, severe), which would have further strengthen the psychometric evidence [5]. As only one parent per child participated, inter‐rater reliability could not be assessed. Although this reflects the reality of home‐based pain management, where one caregiver usually makes the decision, future studies could examine agreement between parents to further strengthen the psychometric evidence. Finally [6], The follow‐up period was limited to two postoperative days, reflecting the expected short duration of pain after minor day‐surgery procedures in children [27]. While appropriate for this context, longer follow‐up may be warranted in studies of more extensive surgery.

### 4.2 Clinical Implications

The PPPM‐S may serve as a helpful tool and complement to support parents in assessing their child's postoperative pain at home. In addition, the PPPM‐S can facilitate communication with healthcare providers by enabling parents to report observable pain behaviours and receive appropriate guidance on pain management. Furthermore, the PPPM‐S may prove useful in future studies on paediatric pain, offering a standardized method for collecting data on postoperative pain experiences in children. Future studies might also explore the PPPM‐S's responsiveness to interventions, such as parental education or analgesic use, as well as its utility in younger children who cannot reliably self‐report using instruments like the CAS.

## 5 Conclusion

The PPPM‐S demonstrated good psychometric properties in this study. Parents reported high satisfaction with the instrument and found it easy to use. This study supports the PPPM‐S as a suitable tool for parents to assess their child's postoperative pain at home. Implementing the PPPM‐S in Swedish paediatric healthcare may ultimately improve postoperative care at home.

Access to the PPPM‐S can be provided upon reasonable request to the corresponding author.

## Author Contributions

C.H. conceptualization; methodology; validation; formal analysis; investigation; data curation; writing original draft; visualization; project administration; funding acquisition. I.T. conceptualization; methodology; validation; writing review and editing; supervision. B.G. conceptualization; methodology; validation; formal analysis; investigation; resources; writing review and editing; supervision.

## Funding

This study was partially funded by the Research and Development Council of Region Östergötland.

## Conflicts of Interest

The authors declare no conflicts of interest.

## Data Availability

The data that support the findings of this study are available from the corresponding author upon reasonable request.
